# A Knockout of the Photoreceptor PtAureo1a Results in Altered Diel Expression of Diatom Clock Components

**DOI:** 10.3390/plants13111465

**Published:** 2024-05-25

**Authors:** Shvaita Madhuri, Bernard Lepetit, Alexander Helmut Fürst, Peter G. Kroth

**Affiliations:** Fachbereich Biologie, Universität Konstanz, 78457 Konstanz, Germany; shvaita.madhuri@gmail.com (S.M.); bernard.lepetit@uni-rostock.de (B.L.); alexander.fuerst@uni-konstanz.de (A.H.F.)

**Keywords:** diatoms, blue light, photoreceptor, circadian clock, mRNA expression analysis, diel regulation

## Abstract

Plants and algae use light not only for driving photosynthesis but also to sense environmental cues and to adjust their circadian clocks via photoreceptors. Aureochromes are blue-light-dependent photoreceptors that also function as transcription factors, possessing both a LOV and a bZIP domain. Aureochromes so far have only been detected in Stramenopile algae, which include the diatoms. Four paralogues of aureochromes have been identified in the pennate model diatom *Phaeodactylum tricornutum*: PtAureo1a, 1b, 1c, and 2. While it was shown recently that diatoms have a diel rhythm, the molecular mechanisms and components regulating it are still largely unknown. Diel gene expression analyses of wild-type *P. tricornutum*, a PtAureo1a knockout strain, and the respective PtAureo1 complemented line revealed that all four aureochromes have a different diel regulation and that PtAureo1a has a strong co-regulatory influence on its own transcription, as well as on that of other genes encoding different blue-light photoreceptors (CPF1, 2 and 4), proteins involved in photoprotection (Lhcx1), and specific bHLH transcription factors (RITMO1). Some of these genes completely lost their circadian expression in the PtAureo1a KO mutant. Our results suggest a major involvement of aureochromes in the molecular clock of diatoms.

## 1. Introduction

Photoautotrophic algae are of major ecological relevance [1,2], representing the base of the aquatic food web. Diatoms are unicellular algae belonging to the Stramenopiles, which are found ubiquitously in both fresh water and in marine systems. These algae represent genetically chimeric eukaryotic organisms. While red algae, green algae, and land plants evolved by uptake of a cyanobacterium that was taken up and converted to an organelle, diatoms evolved by secondary endosymbiosis, which is the uptake of a eukaryotic red alga into a eukaryotic host cell [3,4]. They also display a remarkable capacity to acclimate to changes in environmental conditions [5,6,7,8,9,10].

Light is an essential environmental factor for plants and algae, either as an energy source for photosynthesis or by providing the organisms with information about the surrounding environment [11,12,13]. The effects of fluctuating or oscillating light on algal photophysiology [14,15,16,17] and on competition dynamics are well studied [18,19,20]. Cellular motility can also be affected by specific light conditions, like, for instance, migration of diatoms in sediment mats [21,22] or intracellular plastid migration [23]. Light quality and intensity in these organisms can be sensed by photoreceptors, which usually are pigment-binding proteins that absorb specific wavelengths and which may prompt several cellular responses via signalling cascades or direct regulatory effects. Photoreceptors also have been shown to entrain the circadian clock during diurnal cycles in plants and algae [24,25,26,27].

Blue light is reaching deeper within the water column than other wavelengths of the visible spectrum because of an increased absorption of red light by water. While the ratio of red and far red light can have a strong impact on plant development in land plants [28], the contribution and regulation of red and blue light in diatoms is poorly understood [29]. A peculiar type of algal blue-light receptor was identified in 2007 in the xanthophyte alga *Vaucheria frigida*; it was named Aureochrome [30]. This protein, which also is found in diatoms [31], contains a blue-light responsive flavin-binding LOV domain (Light, Oxygen, Voltage) together with a bZIP (basic leucin zipper) domain, which is a typical domain of bZIP transcription factors [31,32]. Aureochromes have subsequently been detected in other Stramenopiles, but not in other organisms. Interestingly, they are missing in the oomycetes [31,33,34], a non-photosynthetic subgroup of the Stramenopiles [35]. Four paralogues of aureochromes (PtAureo1a, b, c, and 2) were identified in the model diatom *P. tricornutum* [34], with PtAureo1a being the currently best-studied aureochrome both in vivo [34,36,37,38] and in vitro [39,40,41,42]. A transcriptomic study on PtAureo1a knockout mutants revealed that this photoreceptor plays an essential role for a cellular reprogramming after a shift from red light to blue light [43]. The diel expression pattern of individual aureochromes apparently is differently regulated [39]. Therefore, aureochromes are highly interesting candidates for investigating a possible involvement in the regulation of diel biological rhythms of *P. tricornutum*. It was shown that PtAureo1a affects the expression of diatom-specific cyclin (dsCYC2), which is implicated to be important for the G1 to S phase cell cycle transition after dark arrest, and that PtAureo1a is involved in photoacclimation [34,37,44,45].

The diel light−dark cycle is a significant environmental parameter for most organisms. In phytoplankton, it can affect or regulate metabolic processes via the photoperiod, maximum irradiance, spectral composition, or oscillation patterns [17,46]. Microalgae can also show robust diel rhythms in regard to various processes such as growth, gene expression, pigment synthesis, etc., which has been shown in a variety of phytoplankton organisms [27,47,48,49,50]. In the centric diatom *Thalassiosira pseudonana*, about 40% of the transcriptome may change during day and night transitions [51]. In the pennate diatom *Phaeodactylum tricornutum*, it was shown that the cell cycle is following an endogenous circadian pattern, but its synchronization depends on the light−dark cycle [44,52]; however, the molecular mechanism of endogenous circadian clocks in the algae is still unknown. Recently, the RITMO1 protein (PtbHLH1a), a member of the bHLH-PAS transcription factor family, was found to be involved in the regulation of the circadian rhythms in *P. tricornutum* [53]. Overexpression of this protein in this diatom results in a shift of circadian fluorescence patterns. In the well-studied land plant *Arabidopsis thaliana*, various blue-light photoreceptors, such as ZTL (ZEITLUPE) and FKF1 (FLAVIN BINDING, KELCH REPEAT, F-BOX 1), interact with GIGANTEA (GI), a clock-associated protein [54,55,56], controlling processes like photoperiodism. In diatoms, the blue-light receptor PtCPF1 is the only photoreceptor that so far has been shown to be involved in circadian rhythms. It can repress CLOCK:BMAL1-regulated expression of a reporter gene in a heterologous system [57].

Here, we compared expression patterns of a *P. tricornutum* WT strain with the respective PtAureo1a knockout mutant KO8 [37] and the PtAureo1a complemented mutant Co.48 [38], which had been cultivated in photobioreactors under turbidostat conditions. We demonstrated that a circadian expression of specific genes is directly dependent on the presence of PtAureo1a, indicating that aureochrome proteins may play an important role as input signal for the diatom clock.

## 2. Results

### 2.1. Growth Characteristics of WT, KO8 and Co.48

In this study, we compare the diel expression of specific genes of *P. tricornutum* wild-type strain Pt4, the Aureochrome 1a knockout strain PtAureo1aKO8 [37], and a PtAureo1a complementation mutant, PtAureo1aCo.48. PtAureo1aCo.48 is one of a number of mutant lines which have previously been created by complementing a PtAureo1aKO8 strain with the *PtAureo1a* gene driven by its endogenous promoter [38]. PtAureo1aCo.48 expresses the PtAureo1a protein to a very similar extent as the wild-type strain [38] and shows the same phenotype.

**Figure 1:**
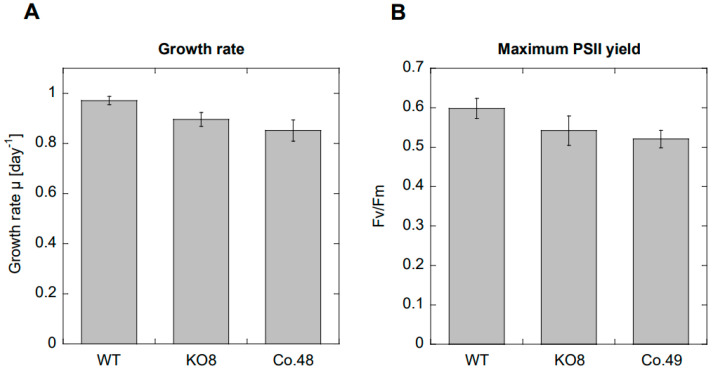
(**A**) Comparison of growth characteristics and (**B**) maximum photosystem II yield (Fv/Fm) of wild-type (WT), PtAureo1a knockout (KO8) and PtAureo1a8−complemented mutant (Co.48) during their exponential phase. Indicated values represent the arithmetic means (*n* = 3 for growth rate, *n* = 5–11 for Fv/Fm), and the error bars represent the standard error. A one-way ANOVA was performed, showing no statistical difference neither for growth (*p* = 0.154) nor for maximum PSII yield (*p* = 0.083) between the three strains.

As a prerequisite for comparing diel and potential circadian gene expression in the selected strains, we studied their growth characteristics. The growth rates during the exponential phase were similar between all three strains (one-way ANOVA, *p* = 0.156, Figure 1A). We furthermore determined the maximum photosynthetic efficiency of photosystem II (PSII) by measuring Fv/Fm of the three diatom strains in order to detect any differences in photosynthetic and metabolic activities. Figure 1B demonstrates that there was no significant difference in the maximum quantum yield of PSII between all three strains (one-way ANOVA, *p* = 0.083).

### 2.2. Diel Regulation of Diatom Genes by PtAureo1a

**Figure 2:**
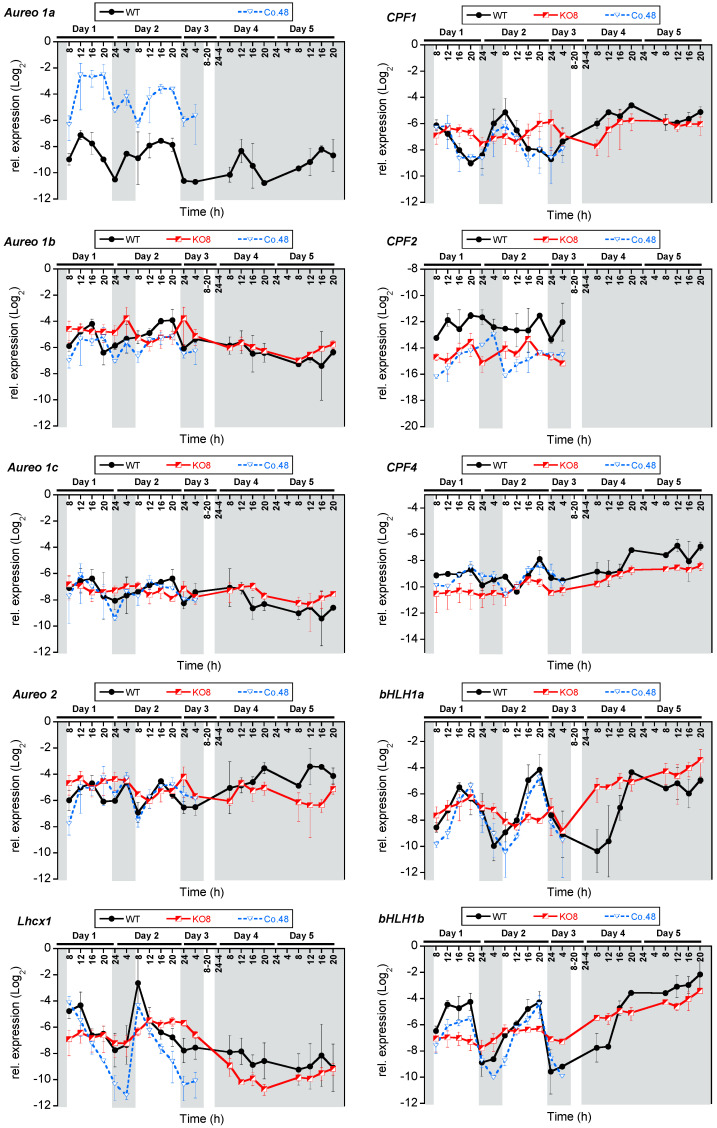
Diel expression of aureochrome genes (*PtAureo1a*, *1b*, *1c*, *2*), cryptochrome photolyase family (*PtCPF1*, *2*, *4*), *Lhcx1*, and *PtbHLH1a*, *1b* transcription factors in *P. tricornutum* WT (black), PtAureo1aKO8 knock out mutant (KO8, red), and PtAureo1a8Co.48−complemented mutant (Co.48, dotted blue line) under 16:8 h L/D and D/D conditions. The relative gene expression is represented as mean Log2 value with SEM (Standard error mean), n = 3 (WT & KO8); n = 2 (Co.48), normalized on *18S*, *TBP* and *RPS*. The white and grey regions represent light and dark periods, respectively. The numbers represent the time points of sampling with respect to each day. Note that the y-scale is equidistant with respect to the relative log2 values for all genes.

Two major factors may limit data quality when studying rhythmic or periodic gene expression patterns in cell cultures: (i) a variable cell density in the samples, and (ii) the degree of culture synchrony that influences the congruity of gene expression patterns related to specific processes, such as cell division. In order to eliminate these factors as much as possible, we have used a turbidostat system for continuous cultivation of the diatoms. Based on optical density measurements, cell densities corresponding to cultures during exponential growth were stabilized via controlled dilution of the culture. Such a dilution with fresh media prevents changes in nutrient concentrations, which is known to affect general gene expression [58]. After 5 days of acclimation to standard conditions (16:8 h light/dark, L/D), we took samples at specific time points for a period of 52 h, each under L/D or continuous darkness (D/D) conditions, and monitored the expression of ten different genes encoding photoreceptors and regulators (PtAureo1a, 1b, 1c, 2, PtCPF1, 2, 4, PtbHLH1a, 1b, and PtLhcx1, listed with accession numbers in Supplementary Table S2) (Figure 2).

*PtAureo1a* transcript abundance (here only the WT and complemented line are studied) peaks 4 h after light onset at Day1 T12/16, and this expression pattern is recurring the next day both in WT and Co.48 (Day2 T12/16), albeit with a higher transcript level in the complemented line. Interestingly, under D/D conditions, the expression of *PtAureo1a* peaks at Day4 T12/16, which is equivalent to Day1 T12/16 in L/D conditions, and this is recurring the next day (Day5 T12/16), implying that the oscillation of *PtAureo1a* expression continues in the dark with no considerable phase shift. Western blot analyses show that the amount of PtAureo1a protein peaks at time points 6 and 24 (Figure S1). In the dark, this fits to a delayed protein synthesis, as there is an increase in transcripts detectable in the middle-of-the-night phase, while the peak of transcription during the day does not result in a direct protein increase, which may be due to light-dependent proteasomal degradation of PtAureo1a [59].

For the other aureochrome genes, the diel mRNA oscillations are weaker but still detectable. The transcript profile of *PtAureo1b* shows a diel regulation during the day, while, in the dark, nearly no induction of transcription is observable, indicating that light is required for induction of transcription. In the PtAureo1a knockout strain, the *PtAureo1b* transcripts show weaker changes, which is indicating that PtAureo1a might be required for the diel expression of PtAureo1b. *PtAureo1c* mRNA oscillation in WT shows a similar pattern as *PtAureo1a*, peaking at Day1 T12, four hours after light onset. Expression of *PtAureo1c* in the PtAureoKO8 mutant line shows a weaker oscillation, which can partially be rescued in PtAureo1aCo48. Transcripts of both *PtAureo1b* and *1c* show a weaker oscillation in the dark phase. Also, *PtAureo2* mRNA expression in WT and PtAureo8Co.48 shows a changed pattern in the knockout mutant, which is in contrast to previous reports showing mostly constant diel expression of *PtAureo2* [39].

We also studied the expression profile of further blue-light photoreceptors belonging to the cryptochrome/photolyase family which supposedly are involved in regulating the clock in diatoms [57]. *PtCPF1* transcription is induced at the beginning of the day (Day1 T8 and Day2 T8), with an mRNA peak one hour after dark to light transition in WT, which is consistent with results reported by [57,60]. In D/D, the transcript expression increased at the same time points as at the beginning of the light phase in L/D, which is abolished in KO8 in the second D/D cycle. The swift increase in expression at the end of the dark period and the endurance of the expression level without light input suggests the ability to anticipate light onset, which is typical for circadian-regulated genes and circadian clock components [57]. A more intriguing observation was a disoriented diel expression of *PtCPF1* in KO8, which was recovered in Co.48 under L/D, indicating a prominent direct or indirect involvement of PtAureo1a in its regulation. *PtCPF2* mRNA is generally only lowly expressed and shows no major differential expression pattern in all strains under L/D. Under constant darkness, no transcripts could be detected, probably due to mRNA abundance below the detection limit. *PtCPF4* mRNA expression shows stable expression throughout the day, with a peak at the end of the light period (Day1 T20 and Day2 T20) in WT and Co.48, while it is feeble in KO8. In constant darkness, there are modest mRNA oscillations in WT which are absent in KO8.

The PtLhcx1 protein provides energy dependent quenching capacity in cells grown under low light conditions [61]. The mRNA oscillation of *PtLhcx1* shows a strong light-dependent diurnal regulation, with an expression peak at Day1 T8 and Day2 T8 both in WT and Co.48. In the KO8 line though, this strong fluctuation is completely abolished, suggesting a regulatory involvement of PtAureo1a in *PtLhcx1* transcription. Under D/D conditions, the oscillation of *PtLhcx1* gene is strongly dampened, in line with previous results [62], implying the importance of light entrainment for regulation of this gene.

The *PtbHLH1a* and *1b* genes show a pronounced and regular diurnal fluctuation of transcript levels in WT. The rhythm is obscured in KO8 but rescued in Co.48. On the first day of constant darkness (Day 4), we observed an increase in *PtbHLH1a* and *1b* mRNA abundance without light induction. This effect is lost the recurring day, while the level of expression remains constantly high in WT. In KO8, this pattern completely disappeared, advocating a direct/indirect involvement of PtAureo1a in regulation of the diel biological rhythm even under dark conditions.

Although, for most genes described here, the wild-type phenotype can mostly be rescued in the complemented mutant, the transcript abundances are not completely identical, which is probably due to a very sensitive response to already minor differences of the Aureochrome 1a protein. Additionally, a weaker rhythm or a reduced oscillation amplitude under continuous darkness could result from the lack of photosynthesis under constant darkness in *P. tricornutum* [53]. To avoid misleading conclusions from the diel expression of the complemented mutant under constant darkness, we did not interpolate the complemented (Co.48) mutant in D/D rhythm analysis.

In order to reveal statistically significant patterns, we analysed the transcript abundances using the BioDare2 software package (https://biodare2.ed.ac.uk/) while calculating Kendal rank correlation coefficients (τ), which describes the ordinal association between two measured quantities. Under L/D conditions, transcript abundances of *PtAureo1a*, *1c*, *PtCPF1* and *4*, *PtbHLH1a* and *1b*, and *PtLhcx1* genes were found to be significantly rhythmic throughout the cycle (shown in Table 1). Here, the Kendall rank coefficients for *PtCPF1*, *PtLhcx1* and both *PtbHLH1* genes exhibited a very high coefficient of >0.8. For D/D, we did not calculate the coefficient, because the chosen sampling intervals were not regular enough.

A lack of PtAureo1a protein in the knockout mutant obviously alters the mRNA oscillation of *PtCPF1*, *PtLhcx1*, and *PtbHLH1a* and *1b* significantly (*p* < 0.01), while rhythmicity is again restored in the complemented mutant (*p* < 0.05).

To further investigate the impact of PtAureo1a only on the significantly diurnally regulated genes with very high Kendall rank coefficients >0.8 *(PtCPF1*, *PtLhcx1*, *PtbHLH1a* and *PtbHLH1b*), we used the arithmetic mean of gene expression to determine the rhythmic parameter period, phase and amplitude by two different methods. The Fast Fourier Transform Non-Linear Least Squares algorithm (FFT-NLLS) is a classical algorithm that tries to fit the sum of cosine functions on a potential regulatory pattern [63]. It has the disadvantage of not recognizing a pattern when the rhythm does not follow a sinusoidal waveform [64]. For the latter, the Maximum Entropy Spectral Analysis (MESA) is better suited, as it does not assume any underlying waveform of the rhythm but is based on stochastic spectrum modelling [64]. Both modelling approaches are suitable also for a relatively short and noisy data series, such as in our case [64].

FFT-NLLS analysis allowed us to fit the data for *PtCPF1*, *PtbHLH1a* and *PtbHLH1b* in WT and Co.48 with a period roughly around 24 h (Table 2). In KO8, no fitting was possible for *PtbHLH1a*. This indicated that the sine pattern of *PtbHLH1a* transcript abundance observed and fitted in WT and Co.49 was lost in the KO8 mutant. Also, in this line the period for *PtCPF1* and *PtbHLH1b* rhythmicity was longer, with >30 h clearly deviating from a circadian period (=24 h). Interestingly, the completely different MESA approach provided similar period estimates as FFT-NLLS analyses for all three genes in all three lines, also in terms of phase (i.e., time of the peak) and amplitude. The latter was strongly dampened in KO8 compared to both WT and Co.48, indicating that PtAureo1a does not only influence the timing of diurnal circulation but also its amplitude.

FFT-NLLS did not fit the rhythm of *PtLhcx1* gene regulation to the sum of cosine functions. Here, we could only rely on MESA analysis. The period estimates were quite distant from 24 h (WT 29.2 h, KO8 27.7 h, Co.48 29.5 h). However, the amplitude was again largely dampened in KO8 compared to WT and the complemented mutant, and a clear phase shift was observed (4.9 h and 6.4 h in WT and Co.49, respectively, versus 10.8 h in KO8). At present, these data indicated an involvement of PtAureo1a in the oscillating daily *PtLhcx1* mRNA pattern, but further and especially longer time series will be needed to provide more robust statistical support. Overall, the FFT_NLLS and MESA analyses supported the results of the Kendal rank analysis: PtAureo1a had a direct or indirect effect on the diurnal mRNA pattern of *PtCPF1*, *PtbHLH1a*, *PtbHLH1b* and *PtLhcx1*.

### 2.3. Correlation Analysis of Diel Expression in P. tricornutum

**Figure 3:**
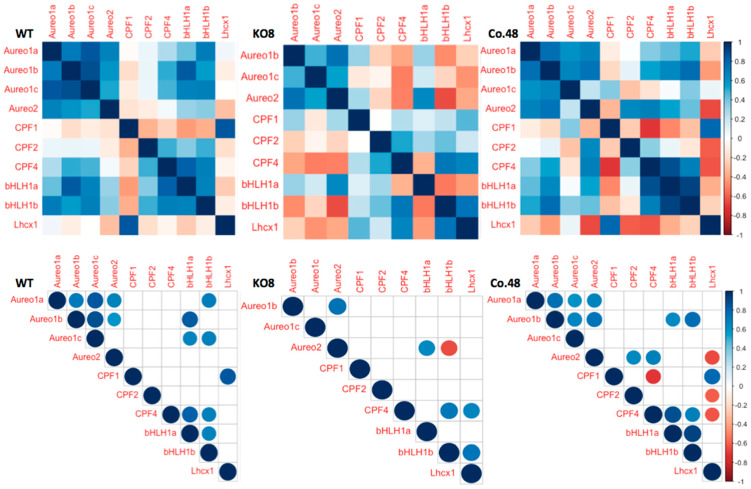
Pearson’s correlation matrix analysis of diel expression under L/D conditions of genes of aureochromes (PtAureo1a, b, c, 2), cryptochrome/photolyase family protein (PtCPF1), Cry−DASH−like family protein (PtCPF2 & 4), PtbHLH−PAS TFs (PtbHLH1a and 1b) and photoprotection protein PtLhcx1 of wild−type (WT), PtAureo1a knockout (KO8) and PtAureo1a−complemented mutant (Co.48): The lower correlograms show correlations with a significance level of an alpha value <0.05 where insignificant correlations are shown as blank boxes in the correlogram. The circle size indicates strength of correlation coefficient, *r* value (small circle ≤ ±0.2; medium circle ≤ ±0.5; normal circle ≤ ±1); for detailed values see Table S3. The matrix analysis was performed using R, packages corrplot and Hmisc (see Section 4).

In addition to the rhythmicity analysis, we performed a correlation analysis with all studied genes in WT cells to elaborate on potential network connections under light/dark and in constant darkness. Figure 3 demonstrates that, under L/D conditions, the positive correlation between diel expression of aureochrome isoforms is significant, while the expression of the *PtCPF1* gene correlates only to the *PtLhcx1* gene. The expression of *PtCPF2* is unrelated to any other tested genes, while *PtCPF4* transcripts significantly correlate to those of *PtbHLH1b*, which again is strongly correlated to *PtAureo1a* and *1c*. PtbHLH1a expression is positively correlated with *PtAureo1b*, *1c*, *PtbHLH1b*, and *PtCPF4*. All the described correlations are not observed in the PtAureo1a knock out mutant (KO8) but are restored in the PtAureo1a complemented mutant (Co.48), indicating that PtAureo1a is an important regulatory factor. Furthermore, the transcripts of the PtAureo2 protein correlate either positively or negatively with *PtbHLH1a* and *1b* expression, respectively, in the KO8 mutant, which is not seen in WT cells, indicating that this regulation is suppressed in the WT cells.

**Figure 4:**
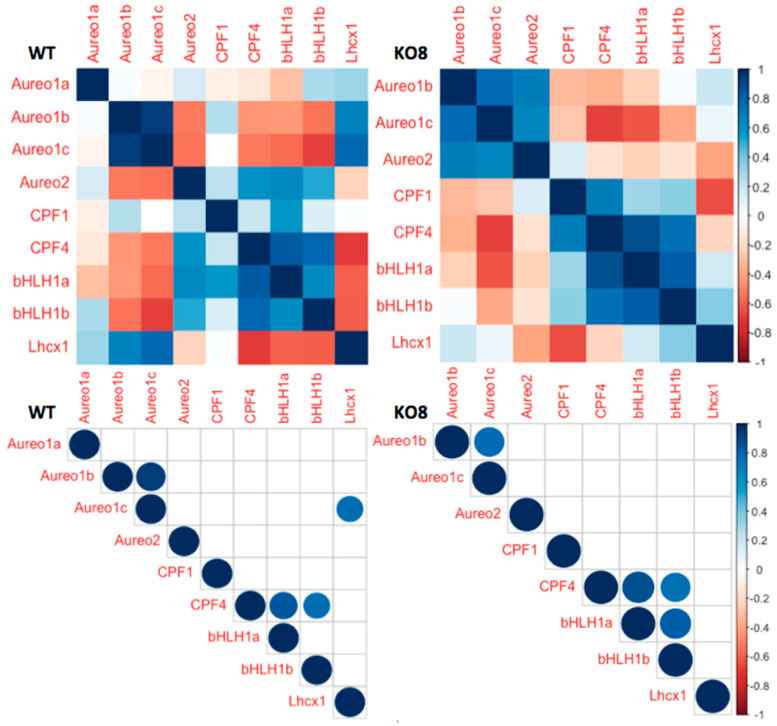
Pearson’s correlation matrix analysis of diel expression of genes of aureochromes (PtAureo1a, b, c, 2) under D/D conditions; cryptochrome/photolyase family protein (PtCPF1), Cry−DASH-like family protein (PtCPF2 & 4), bHLH−PAS TFs (PtbHLH1a and 1b) and photoprotection protein PtLhcx1 of wild−type (WT) *P. tricornutum* and PtAureo1a knock out mutant (KO8) (Upper). Correlogram showing correlation with significance level of an alpha value < 0.05 where insignificant correlations are left with blank boxes in the correlogram (lower figures). The circle size indicates strength of correlation coefficient, *r* value (small circle ≤ ±0.2; medium circle ≤ ±0.5; normal circle ≤ ±1); for detailed values see Table S3. The matrix analysis was performed using R, packages corrplot and Hmisc (see Section 4).

In D/D conditions, PtAureo1b and 1c expression is positively correlated in WT and KO8, independent of presence/absence of PtAureo1a (Figure 4). *PtAureo1c* transcript abundance is strongly correlated with that of *PtLhcx1* in constant darkness in WT, but not in KO8. *PtbHLH1a* and *1b* are found to be not correlated with each other in WT but positively correlated in KO8 under constant darkness.

## 3. Discussion

Biological rhythms, including diurnal regulation of gene expression, have been described for different algal taxa and for different environments [50,65], while the mechanisms of algal clocks are largely unknown. Auto-regulatory transcription−translation regulatory feedback loops (TTFLs) that result in an oscillation of mRNA and proteins are key components of biological clocks [66]. One potential regulator of TTFLS can be light, which is perceived by photoreceptors, that may regulate or adjust the biological clock in plants [67] and green algae [68]. Even non-photosynthetic organisms like fungi may have such TTFLs, involving the White Collar Complex (WCC) photoreceptors of the fungus *Neurospora crassa* [69]. However, the genes encoding TTFL components lack a genetic conservation across the kingdoms [46], making it difficult to identify them. TTFLs in fungi, animals, and plants often comprise proteins that post-translationally modify other proteins, including kinases and methyltransferases—examples are casein kinases, glycogen synthase kinase 3, and protein arginine-N-methyltransferase [70,71,72]. For diatoms, no orthologues of bacterial, animal, or plant circadian clock components have yet been found in the diatom genomes, except for CRYs and casein kinases [73], although the uptake of silicic acid and the general photophysiology [48] of diatoms depend on the diel cycle. The cell division of *P. tricornutum* is regulated by blue light, which is hypothesised to be the mechanism for synchronized cell division under light−dark cycles [44]. The recent reports on proteins such as bHLH-PAS (basic Helix-Loop-Helix-Per/Arnt/Sim) proteins, like RITMO1 (PtbHLH1a), that possess domain structures that are also found in clock components of animals [53,74], now open up the possibility to identify potential interaction partners. RITMO1 displays a robust diel gene transcript pattern, which is adjusted in a photoperiod-dependent manner, and the rhythmic expression persists in cells exposed to continuous light. The gene for the related PtbHLH1b shows a similar expression pattern as RITMO1 in a light−dark cycle, and thus might have a similar function [53]. Our data show that the genes of both PtbHLH1a and 1b proteins show a rhythmic expression in the light that persists in the dark at least for one day. *PtbHLH1a* and *1b* genes lose their diel expression patterns in the PtAureo1a knockout mutant, which can be rescued in the PtAureo1a complemented mutant in LD conditions (Figure 2). Together with the finding that a knockout of PtAureo1a has a strong effect on the two PtbHLH proteins during a shift from red to blue light [43], it suggests that PtAureo1a is involved in the blue light-dependent regulation of RITMO1 and, thus, the diatom circadian clock.

It has been proposed that blue light photoreceptors may play a key role for aquatic photosynthetic organisms [49], because blue light penetrates deeper into the water column than light of longer wavelengths. In a metagenomic approach, Coesel et al. studied the diel transcription of genes in marine algae and found that, among Stramenopiles, the genes of aureochromes and other photoreceptors may respond to and anticipate fluctuating light conditions [50]. Therefore, we studied the diel gene expression of other blue light photoreceptors, i.e., cryptochrome/photolyase family 1 (CPF1) and Cry-DASH-like CPF (2 & 4) [60], and the role of PtAureo1a in regard to their expression. Consistent with Oliveri et al. [60], we found that all CPF transcripts peak during the day, but at different times and with different intensities. Our finding that the clear diel expression pattern of *CPF1* is lost in the PtAureo1a knockout mutant and rescued in PtAureo1a complemented lines is indicative for a direct or indirect effect of PtAureo1a on CPF gene expression.

The demonstration that, in PtAureo1a KO mutants, the expression of a very large number of genes is affected as a response to both a shift from red to blue light [43] and also the diel expression (this study) may, in part, be attributed to the fact that aureochromes, in contrast to other photoreceptors, are both photoreceptors and transcription factors. This allows them to transmit their light responses directly to gene expression, avoiding intermediate phosphorylation steps of further messenger proteins. Accordingly, a strong effect on gene expression in response to blue light can already be observed after less than 10 min [43]. While a knockout of PtAureo1a may have a positive effect on the expression of PtCryP and a larger set of other photoreceptors [43], silencing of the *PtCryP* gene itself only changes the gene expression of diatom phytochrome PtDPH and PtCPF1 [75], again indicating a prominent role of PtAureo1a in the regulation of diel expression. We observed a significant correlation between the diel expression of the aureochrome isoforms with that of the PtbHLH1a/1b TFs. Specifically, the matrix analyses revealed a compelling correlation of *PtAureo1a* and *1b* with *PtbHLH1a*/*1b* genes, as well as *PtAureo1c* under L/D conditions in WT, which is only detected in the presence of PtAureo1a. Taken together, our data indicate that PtAureo1a may control or adjust the diatom clock by controlling the diel expression of *PtbHLH1a*/*1b*. Furthermore, the expression of the gene of the flavin-lacking PtAureo2 protein correlates under L/D conditions with the *PtbHLH1* genes only in the KO8 line, which indicates that PtAureo1a may function as a repressor of PtAureo2 diel expression.

As the most prominent known phenotype of PtAureo1a mutants is a change in photoacclimation [34,37,38], we studied the impact of PtAureo1a on the expression of the gene of the photoprotective PtLhcx1 protein [61]. Indeed, we could observe that the strongly light-dependent diel expression pattern of *PtLhcx1* observed in WT and Co.48 lines is mostly absent in the PtAureo1a KO mutant. At the same time, we do not have any indication yet that Lhcx1 itself might be involved in clock regulation, but rather may be light-induced to facilitate dynamic tracking of light fluctuations in turbulent waters [76]. In line, beyond its control via PtAureo1a, *PtLhcx1* expression responds to light intensity changes transduced via the redox state of the plastidic plastoquinone pool [62].

**Figure 5:**
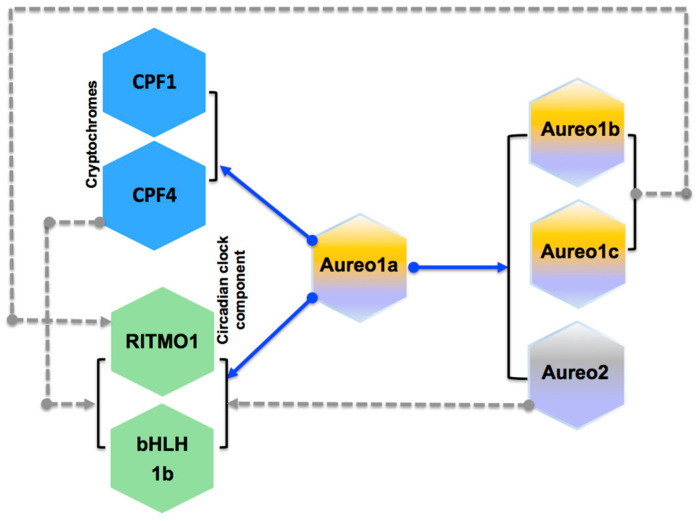
Scheme depicting and summarizing the obtained results on correlated expression of genes and the dependence in PtAureo1a, including the other aureochrome isoforms (PtAureo1b, c and PtAureo2), cryptochromes (PtCPF1 and 4) and PtbHLH1a/b). PtAureo1a might directly/indirectly influence the expression of the diatom clock component RITMO1 as well as the photoreceptors PtCPF1 and PtCPF4. Blue lines represent results from biological experiment (qPCR analysis), while grey dotted line represent results from Pearson’s correlation analysis (statistical).

As a conclusion, our results indicate that PtAureo1a may be an important light-driven regulator of rhythmic gene expression in diatoms (Figure 5). In land plants, the time point of the transcript peaks can be very important for the regulation of clocks, as it reflects the synthesis of the respective proteins and its regulatory activity shortly thereafter. Coesel et al. [50] demonstrate that, among diatoms and related algal groups, individual aureochrome genes may be transcribed at night, as well as in the morning and up to noon, indicating that the aureochromes are ready as soon as light is available, stressing the role of aureochromes as “early” regulators. In *P. tricornutum*, we found the different aureochrome transcripts peak during the day, with PtAureo1a being the first of them being expressed. The investigations on PtAureo1a may represent an entrée into understanding the diatom clock and revealing unknown components of the clock in the environmentally important group of algae that evolved by secondary endosymbiosis. Future work will therefore have to focus on the subsequent processes initiated by PtAureo1a.

## 4. Materials and Methods

### 4.1. Experimental Set-Up and Growth Conditions

*P. tricornutum* (Bohlin) wild-type strain UTEX646 (Pt4) was initially obtained from the culture collection of algae of the University of Texas (UTEX, Austin, TX, USA, https://utex.org/). The TALEN mediated knockout strain PtAureo1aKO8 had been produced by [37], while its complemented strain PtAureo1aCo.48 is described in [38]. The cultivation for the day/night cycle experiments was carried out in photobioreactors (PBR102-S, Phenometrics, Inc., East Lansing, MI, USA) containing up to 400 mL of F/2 medium without added silica and at 50% sea water concentration medium. Cultures were grown at 20 °C with bubbling by using a mini diaphragm pump (VP86, VWR International Ltd., Lutterworth, UK), as well as by magnetic stirring at 300 rpm. The pH of the medium was adjusted to 7.4 before the start of the experiment using 1N HCl/NaOH. The white LED light (resembling full sunlight spectrum) was programmed to provide a continuous light of 100 μmol photons m^−2^ s^−1^ in a 16:8 h L/D cycle (light onset at 7 a.m. and light offset at 11 p.m.). For the continuous dark condition (D/D), the lights were programmed to switch off after acclimation to a 16:8 h L/D cycle. The cultures were maintained in their logarithmic phase at a Chl *a* concentration of approximately 1 μg/mL by regular automatic measurement of the optical density and accordingly variable dilution with fresh F/2 medium by the Phenometrics turbidostat pumps. Cells were acclimated to these conditions for at least 5–7 generations (5–7 day−night cycles) before sample collection for the experiment. Samples were harvested after dawn, starting at Day1 T8 (8 a.m.) in 4 h intervals until the 3rd day before dawn, i.e., at Day3 T4. Notably, 30 mL samples were taken under sterile conditions, frozen in liquid nitrogen and stored at −80 °C for further analyses. After the regular light−dark incubation, another 16:8 h light−dark cycle was added to allow cultures to reach sufficient cell numbers after sampling before switching off the light during the subjective day photoperiod. During the dark phase, samples were harvested similarly.

Along with the wild-type, the TALEN mediated knockout mutant strains PtAureo1aKO8 of *P. tricornutum* [37] and the complemented strain PtAureo1aCo.48 [38] were grown axenically in liquid F/2 medium without added silica and at 50% sea water concentration. Cells in liquid F/2 medium were cultivated in a 16 h/8 h light/dark cycle in Erlenmeyer flasks under continuous shaking at 20 °C and with an illumination of 45 μmol photons m^−2^ s^−1^ (Osram Lumilux L58W/840, Munich, Germany).

### 4.2. Determination of Growth Rates

Cell numbers were counted using a Multisizer 4 Coulter Counter (Beckman, Brea, CA, USA) and the growth curve experiment was set up with an equal number of cells (100,000 cells/mL). Cell number was measured over a period of 10 days in the bioreactor and without diluting with fresh medium to observe growth until the stationary phase.

The growth rate (µ) was calculated during the exponential phase using the following formula:

$$\frac{\mu =\ln\left(N_{t}\right)-\ln\left(N_{0}\right)}{\Delta t}$$

with:

ln N_t_ = Natural Logarithm of cell no. at day 5;
ln N_0_ = Natural logarithm of cell no. at day 3.

Δt = t_1_ − t_0_

### 4.3. Chlorophyll Fluorescence Measurements

Using an AquaPen-C AP100 (Photon Systems Instruments, Drásov, Czech Republic), mid-exponential phase diatom cultures were analysed to determine the maximum photosynthetic efficiency of PSII via the F_v_/F_m_ ratio [F_v_/F_m_ = (F_m_ − F_0_)/F_m_], where F_m_ = maximum fluorescence measured in very low light adapted (30 min) cells induced by a saturating blue light pulse of 2100 μmol photons m^−2^ s^−1^ and F_0_ = minimum fluorescence of low light adapted cells.

### 4.4. RNA Isolation and cDNA Synthesis

RNA was isolated using Peqgold RNAPure and Peqgold Total RNA Kit S-Line with an on-column DNA digest using the Peqgold DNase I Digest kit (VWR, Darmstadt, Germany). RNA was extracted from samples normalized to 1 µg/mL Chlorophyll *a*, and concentration was measured spectrophotometrically using a Nanodrop 2000 UV/VIS Spectrophotometer (Thermo Fisher, Schwerte, Germany). Additionally, cDNA synthesis was performed using the Primescript kit (Takara Bio Europe, Saint-Germain-en-Laye, France).

### 4.5. Quantitative Real-Time PCR (qPCR)

We analysed the transcript abundance of 10 genes: *PtAureo1a*, *PtAureo1b*, *PtAureo1c*, *PtAureo2*, *PtCPF1*, *PtCPF2*, *PtCPF4*, *PtbHLH1a*, *PtbHLH1b*, and *PtLhcx1* using HPLC grade primers (specified in Supplementary Tables S1 and S2) by qPCR. Additionally, 18S, TBP (TATA Binding Protein) and RPS (ribosomal protein S1) were used as reference genes for qPCR analysis. qPCR was run on a 7500 Fast RT-PCR system (Applied Biosystems, Waltham, MA, USA) and, for technical reasons, a few samples were run on CFX Connect^TM^ Real-Time system (Bio-Rad, Hercules, CA, USA). There were no significant differences between the obtained data from the two different devices. Samples were measured in biological triplicates as well as technical duplicates for each gene. Ct values (cycle threshold) and gene amplification efficiencies were calculated using PCR Miner 4.0 [77] from the obtained raw data. Relative mRNA transcript levels were calculated according to [78].

### 4.6. Analysis of Circadian Rhythm and Its Parameters

Circadian rhythm analysis was performed on time-series data using the online tool BioDare2 (https://biodare2.ed.ac.uk/) [64], based on the empirical JTK_CYCLE method [79]. The Kendal rank correlation coefficient (τ) was calculated in the range of [−1,1], where, τ = +1 implies a perfectly correlated series score, τ = −1 implies an anti-correlated series score, while τ = 0 implies an uncorrelated series score. The scores obtained were further optimized by applying a Benjamini−Hochberg correction (BH-factor) to reduce the false positive discovery rate (FDR). Further, a representative circadian rhythm curve is depicted in a particular measure that varies according to time. This difference between the crest and trough values is the amplitude of the rhythm. The timing of a reference point in the cycle (e.g., the peak) relative to a fixed event, such as the beginning of the night phase, is the phase parameter of the rhythm. In our case time point 0 h at midnight was chosen as the fixed event. The time difference between two peaks is called the period. Thereby, the parameters of circadian rhythm, i.e., amplitude, phase, and period, were calculated using two different methods available in the BioDare2 online tool [64]: 1. The Fast Fourier Transform Non-Linear Least Squares algorithm (FFT-NLLS), an algorithm that fits the sum of cosine functions on a sinusoidal time pattern; and 2. MESA (Maximum Entropy Spectral Analysis), an algorithm that applies a stochastic spectrum modelling approach.

### 4.7. Correlation Analysis

We used statistical tool “R” and the packages “corrplot” and “Hmisc” to perform the Pearson product moment correlation coefficient analysis (http://www.R-project.org/) of tested genes in order to hypothesize their diel expression network (script in the Supplementary Figure S2).

## Figures and Tables

**Table 1:** Rhythm analysis of transcript abundances determined via qRT-PCR by amplifying transcripts of PtAureochromes (*1a*, *b*, *c* and *2*), PtCPF blue-light photoreceptors (*1*, *2* and *4*), bHLH-PAS proteins (*PtbHLH1a* and *b*), and the PtLhcx1 gene from WT, KO8, and Co.48 cultivated in photobioreactors under L/D conditions. Kendal rank correlation coefficients (τ) were calculated using empirical JTK_CYCLE method from the online tool BioDare2. τ = 1 would state a perfectly correlated time-series. Statistical significance (*p*-value) is represented as * < 0.1; ** < 0.05; *** < 0.01; **** < 0.005 and ***** < 0.001 with Benjamini−Hochberg correction.

Proteins and Role	Gene Name	Kendall Rank Correlation Coefficient (τ)		
		L/D		
		WT	KO8	Co.48
Aureochrome Isoforms	*PtAureo1a*	0.64 *		0.66 *
	*PtAureo1b*	0.59	0.36	0.71 **
	*PtAureo1c*	0.73 **	0.52	0.78 **
	*PtAureo 2*	0.54	0.4	0.64 **
Cryptochrome/photolyase family protein1	*PtCPF1*	0.81 ****	0.4	0.74 **
Cry-DASH-like protein family	*PtCPF2*	0.46	0.59	0.72 **
	*PtCPF4*	0.64 *	0.56	0.83 ****
Circadian clock-related	*PtbHLH1a* (*Ritmo1*)	0.88 ****	0.42	0.92 *****
	*PtbHLH1b*	0.88 ****	0.46	0.89 ****
Photoprotection	*PtLhcx1*	0.87 ****	0.38	0.9 ****

**Table 2:** Period (h), phase (h) and amplitude analysis of *PtCPF1*, *PtLhcx1*, *PtbHLH1a* and *1b* in WT, KO8 and Co.48 under 16:8 h (L/D). The BioDare2 online tool was used to apply FFT-NLLS and MESA analyses to calculate period and phase estimates.

		Period		Phase		Amplitude	
		*FFT-NLLS*	*MESA*	*FFT-NLLS*	*MESA*	*FFT-NLLS*	*MESA*
*CPF1*	WT	26.3	26.1	5.4	5.7	1.5	1.5
	KO8	31.4	34.2	10.6	8.7	0.6	0.7
	Co.48	24.3	25.1	7.6	6.7	1.1	1.1
*bHLH1a*	WT	25	24.8	16.6	16.8	2.3	2.2
	KO8	no fit	26.7	no fit	18.3	no fit	0.6
	Co.48	23.4	23.8	19.9	19.3	2.4	2.3
*bHLH1b*	WT	24.4	24.9	15.4	14.8	2.2	2.1
	KO8	31.5	25.6	5.2	11.2	0.5	0.4
	Co.48	25.3	25.3	15.1	15	2.4	2.3
*Lhcx1*	WT	no fit	29.2	no fit	4.9	no fit	1.3
	KO8	no fit	27.7	no fit	10.6	no fit	0.5
	Co.48	no fit	29.5	no fit	6.4	no fit	2.1

## Data Availability

Data are contained within the article and Supplementary Materials.

## Supplementary Materials

The following supporting information can be downloaded at: https://www.mdpi.com/article/10.3390/plants13111465/s1, Table S1: Primers used in the study for RT-qPCR describing respective target genes and sequences, Table S2: JGI Protein IDs and Ensembl IDs of genes investigated in this study; Table S3: Pearson’s correlation coefficient (*r*) analysis of diurnal rhythm of test genes used in this study between 3 groups as: WT and *PtAureo1a* knock out mutant (KO8); KO8 and *PtAureo1a* complemented mutant (Co.48); WT and Co.48 under L/D condition; Figure S1: Western blot showing the diurnal abundance of PtAureo1 protein in *P. tricornutum* WT cells using anti-PtAureo1a antiserum and, as a loading control, D1 antiserum. Figure S2: R Script for Correlation analysis. R script used in this study to perform Correlation analysis on 10 test genes mRNA expression data under D/D condition for WT.
