# Supplementary material for: A Knockout of the Photoreceptor PtAureo1a Results in Altered Diel Expression of Diatom Clock Components
Source: Plants (Basel). 2024 May 25;13(11):1465. doi: 10.3390/plants13111465 (PMC11174801; doi:10.3390/plants13111465)
Supplement: Supplementary file 1 [file plants-13-01465-s001.zip › plants-2842665-supplementary.pdf]

SUPPLEMENTARY DATA

Table S1. Primers used in the study for RT-qPCR describing respective target genes and sequences.

Target gene	qPCR Primer	Sequence 5'->3'
PtAureo1a	Aureo1a for.	CCACCACCACCAACACTAGG
	Aureo1a rev.	AGGCGTGAACATGACGTCT
PtAureo1b	Aureo1b for.	GCTGTACCTCGAGTCCGTTG
	Aureo1b rev.	AAGCATTGCTGTCCCATTGTC
PtAureo1c	Aureo1c for.	ACGTTGCGTCCACACACATA
	Aureo1c rev.	GTCGTCGAGATCCAGAGTGG
PtAureo 2	Aureo 2 for.	GTTCTCAAGGAGCTGGTCAAG
	Aureo 2 rev.	AACGAGTGTTGGGAACTCTGG
PtCPF1	CPF1 for.	TTCTTGCACTGCCCATTTCT
	CPF1 rev.	CATGTCCTTGAACTGAGGCA
PtCPF2	CPF2 for.	TCCTTCCGCCTTGAAACA
	CPF2 rev.	CTCGTCATGGGTTTCGACTT
PtCPF4	CPF4 for.	GACTAGCCCATCCCAAACAG
	CPF4 rev.	CCTTTGAATCGAGCAGTCG
PtbHLH1a	bHLH1a for.	CGACCATGAGTGATTTTCAGTTT
	bHLH1a rev.	ACGTGGACGATTCCGAACC
PtbHLH1b	bHLH1b for.	GCAATTGCCTGTAAACACGGT
	bHLH1b rev.	TGTTGTTGACTATTGATGGCTCC
PtLhcx1	Lhcx1 for.	CAAACAAGCCGTGCGTC
	Lhcx1 rev.	CGAATCCAAGAGGGTCGAA
18S	18S for.	AAGTTCTCGCAACCAACAC
	18S rev.	CGCCTCAATCAAGGTCAAGAT
PtRPS	RPS for.	AATTCCTCGAAGTCAACCAGG
	RPS rev.	GTGCAAGAGACCGGACATAC
PtTBP	TBP for.	ATCGATTTGTCAATCCACGAG
	TBP rev.	ATACAGATTCTGTGTCCACGG

Table S2. JGI Protein IDs and Ensembl IDs of genes investigated in this study.

	**JGI Protein ID**	**Ensemble ID**
PtAureo1a	49116	Phatr3_J8113
PtAureo1b	49458	Phatr3_J15977
PtAureo1c	56742	Phatr3_J51933
PtAureo 2	56060	Phatr3_J15468
PtCPF1	27429	Phatr3_J27429
PtCPF2	34592	Phatr3_J34592
PtCPF4	55091	
PtbHLH1a		Phatr3_J44962
PtbHLH1b		Phatr3_J44963
PtLhcx1	27278	Phatr3_J27278
18S		EMLSAT00000013183
PtRPS	45451	
PtTBP	10199	

Table S3. Pearson's correlation coefficient (*r*) analysis of diurnal rhythm of test genes used in this study between 3 groups as: WT and *PtAureo1a* knock out mutant (KO8); KO8 and *PtAureo1a* complemented mutant (Co.48); WT and Co.48 under L:D condition. The correlation was calculated using Sigmaplot version 14. The pair(s) of variables with positive correlation coefficients and *p*-values below 0.05 tend to increase together. Statistical significance (*p*-value) is represented as * < 0.1; ** < 0.05; *** < 0.01; **** < 0.005 and; ***** < 0.001.

S.No.	*Gene Name*	*WT and KO8*		*KO8 and Co.48*		*WT and Co.48*	
		r-value	*p*-value	*r*-value	*p*-value	*r*-value	*p*-value
1	*Aureo1a*					0.655^{**}	0.02
2	*Aureo1b*	-0.406	0.19	-0.215	0.503	0.606^{**}	0.03
3	*Aureo1c*	-0.265	0.405	0.0481	0.882	0.702^{**}	0.01
4	*Aureo 2*	0.0483	0.881	0.299	0.344	0.535^{*}	0.07
5	*CPF1*	-0.384	0.217	-0.211	0.511	0.815^{****}	0.001
6	*CPF2*	-0.0149	0.965	-0.038	0.912	0.238	0.457
7	*CPF4*	0.414	0.181	0.494	0.102	0.67^{**}	0.017
8	*bHLH1a*	-0.0389	0.904	0.251	0.431	0.884^{*****}	0.0001
9	*bHLH1b*	0.361	0.248	0.562	0.0569	0.849^{*****}	0.0004
10	*Lhcx1*	-0.0418	0.897	-0.16	0.619	0.744^{***}	0.005

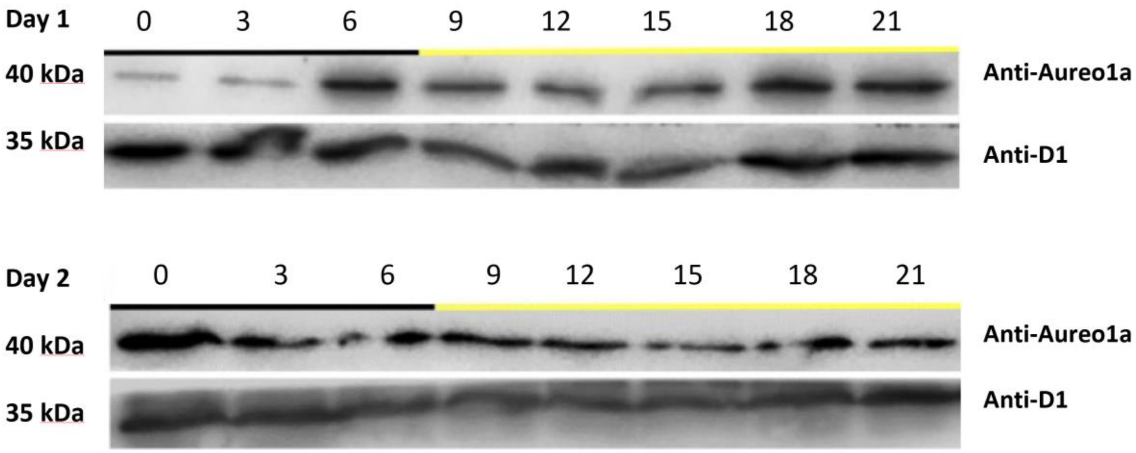

Fig. S1. Western blot showing the diurnal abundance of PtAureo1 protein in *P. tricornutum* WT cells using anti-PtAureo1a antiserum (upper lines) and, as a loading control, D1 antiserum (lower lines), labelling the D1 protein of photosystem II. The time index is shown as daily time points of sampling starting at midnight. The expected molecular weight of Ptaureo1a is 41,5 kDa. The black line indicates darkness, the yellow line light conditions. The protein abundance follows the transcript abundance shown in Fig. 2 with a certain delay.

Material and Methods - Western blot

Cell pellets corresponding to approximately 150 million cells were resuspended in lysis buffer (4 M urea, 1.5 M thiourea, 1% SDS, 20 mM Tris pH 7.5) and 1X protease inhibitor (Complete EDTA-free, Roche, Basel, Switzerland) for protein extraction. A small spatula full of 1 mm, 0.5 mm and 0.1mm diameter beads each was added and the cells were lysed in a Savant FastPrep FP120 bead mill (Thermo Scientific, Karlsruhe, Germany) six times for 20 s with intermittent cooling on ice. Cell debris and residual beads were removed by centrifugation at 18,000 g, 4 ^{o}C for 60 min. Separation of proteins was done in a 12% polyacrylamide gel by SDS-PAGE (Laemmli, 1970). After blotting using a Trans-Blot Turbo Transfer System (Biorad, Hercules, California, United States), the nitrocellulose membrane (Amersham Protran 0.1 µm NC, GE Healthcare, Fisher Scientific GmbH, Schwerte, Germany) was blocked with Roti-Block (Carl Roth, Karlsruhe, Germany) overnight. Next, it was cut between 35 and 40 kDa. The top half (40-250 kDa) was incubated with custom-made antiserum specific against *PtAureo1a* (Agrisera AB, Vännas, Sweden) diluted 1:1000 and the bottom half (10-35 kDa) with D1-specific antiserum (AS05-084, Agrisera AB) diluted 1:10000 as a loading control for 1hr. After incubation with the primary antibody, blots were incubated with 1:10000 dilutions of goat anti-rabbit IgG (H&L) HRP conjugate secondary antibody (AS09 602, Agrisera AB) for 1hr.

```r
View(WT.DD)
attach(WT.DD)
library("corrplot")
library("Hmisc")
cor1<- rcorr(as.matrix(WT.DD))
M<- cor1$r
p_mat<- cor1$P
corrplot(M, type = "full", p.mat = p_mat, sig.level = 0.05)
corrplot(M, type = "upper", p.mat = p_mat, sig.level = 0.05, insig = "blank")
corrplot(M, type = "upper",order = "hclust", p.mat = p_mat, sig.level = 0.05, insig = "blank")
```

Fig. S2 R Script for Correlation analysis

R script used in this study to perform Correlation analysis on 10 test genes mRNA expression data under D:D condition for WT.
